# Knockdown of SP1/Syncytin1 axis inhibits the proliferation and metastasis through the AKT and ERK1/2 signaling pathways in non‐small cell lung cancer

**DOI:** 10.1002/cam4.2448

**Published:** 2019-08-09

**Authors:** Xiaohui Li, Yang Fu, Xiyan Xia, Xin Zhang, Ke Xiao, Xuewei Zhuang, Yi Zhang

**Affiliations:** ^1^ Department of Clinical Laboratory Medicine Shandong University Qilu Hospital Jinan China; ^2^ Jinan Maternity and Child Care Hospital Jinan China; ^3^ Jinan‐Vocational College of Nursing Jinan China

**Keywords:** apoptosis, cell cycle, non‐small cell lung cancer, proliferation, SP1, Syncytin1

## Abstract

Syncytin 1 is considered as an oncogene in various malignant tumors, but its effect on non‐small cell lung cancer (NSCLC) has not been reported. We investigated the specific role of Syncytin 1 on NSCLC through the transfection of Syncytin 1 knockdown or overexpression plamids in A549 cells. Our results proved that knockdown of Syncytin 1 inhibited the proliferation, and blocked the cell cycle on G1 phase by inhibiting the expression of Nusap1, Cyclin D1, CDK6, and CDK4. Cell cycle arrest also leaded to increased apoptosis in Syncytin 1 knockdown cells. Suppression of Syncytin 1 inhibited the migration and invasion, as well as the expressions of epithelial‐mesenchymal transition (EMT) makers, N‐cadherin, β‐catenin, and Vimentin, indicating that Syncytin 1 knockdown inhibited the metastasis via reversing the EMT process in A549 cells. The phosphorylation levels of Akt, mTOR, and Erk1/2 were all decreased in Syncytin 1 knockdown cells, suggesting the signaling pathways by which Syncytin 1 operated as an oncogene in NSCLC. Moreover, the underexpression of transcription factor SP1 downregulated the Syncytin 1 expression in A549 cells. The rescue experiment of Syncytin 1 in SP1 knockdown cells further proved that Syncytin 1 could block the inhibition of cell growth induced by SP1 knockdown. In conclusion, knockdown of SP1/Syncytin1 axis inhibited the progression of NSCLC by the reversion of tumor epithelial‐mesenchymal transition process and suppression of Akt and Erk signaling pathways, suggesting that they are potential targets for targeted therapy of NSCLC.

## Graphical abstract

We investigated the specific role of Syncytin 1 on non‐small cell lung cancer (NSCLC) through the transfection of Syncytin 1 knockdown or overexpression plamids in A549 cells. Knockdown of SP1/Syncytin1 axis inhibited the progression of NSCLC by the reversion of tumor epithelial‐mesenchymal transition process and suppression of Akt and Erk signaling pathways, suggesting that they are potential targets for targeted therapy of NSCLC.

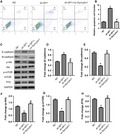

## 1 INTRODUCTION

Lung cancer is one of the most common malignant tumors, and the incidence and mortality of lung cancer worldwide has increased year by year, especially in developing countries. Non‐small cell lung cancer (NSCLC) accounts for about 80% of all lung cancers.^1^ At present, the prevention and treatment of NSCLC has become a widespread concern worldwide. Moreover, in recent years, the treatment of NSCLC has made considerable progress, especially the molecular targeted therapy, including EGFR, PD‐L1, ALK, and anti‐angiogenesis inhibitors.^2^, ^3^, ^4^, ^5^ The present research aimed to explore the molecular mechanisms and to provide a new molecular therapeutic target for NSCLC.

Syncytin1 is the envelope protein cytokine of human endogenous retrovirusesW1 (HERVW1), and its coding gene is located on human chromosome 7q21.2, which is a gene trace after HERVW1 infects human germ cells.^6^ Human endogenous retroviruses (HERVs) are relics of the infective retrovirus integrated into the human genome, accounting for approximately 5%‐8% of the human genome, including at least 31 families.^7^ They participate in the regulation of various physiological and pathological activities in humans through different pathways.^8^, ^9^ Most of the HERVs genes lose their transcriptional ability due to mutation or partial deletion, but some genes remain intact as open reading frames and can be translated into functional proteins, such as Syncytin 1. Studies have proved that Syncytin1 plays an important role in fetal development, and its abnormal expression can lead to intrauterine growth retardation, pathological embryos, tumors, and multiple sclerosis.^10^, ^11^, ^12^, ^13^, ^14^

Although Syncytin 1 has been studied in breast, endometrial, melanoma, colorectal, ovarian, testicular, and hematological tumors, its mechanism is still unknown, and the role of Syncytin 1 in lung cancer has not been reported. Therefore, we investigated the specific role and molecular mechanism of Syncytin 1 on NSCLC to provide a potential target for the clinical treatment of human NSCLC.

## 2 MATERIALS AND METHODS

### 2.1 Cell culture and transfection

The NSCLC cell line A549 used in this research was purchased from the Type Culture Collection of the Chinese Academy of Sciences. Cells were grown in DMEM medium supplemented with 10% FBS and 1% penicillin and streptomycin combination. Vectors with Syncytin 1 shRNAs or overexpression sequences and vector with SP1 shRNAs were purchased from Genechem Co., Ltd. The transfection was performed using Lipofectamine 2000 (Thermo Fisher Scientific) when cells reached approximately 70% confluency. The cells were incubated under normal conditions at 37°C with 5% CO_2_.

### 2.2 RNA extraction and quantitative real‐time PCR

Total RNA from A549 cells were extracted using trizol reagent (Thermo Fisher Scientific), and then reverse transcribed to cDNA. The expression of lncOGFRP1 was performed by SYBR Premix Ex Taq II. The relative quantification was identified by the 2^−∆Ct^ method after standardization to the β‐actin level. Primers used in this research were listed as follows: Syncytin‐1, forward 5′‐CGCCAATGCCAGTACCTAGT‐3′ and reverse 5′‐TCATATCTAAGCCCCGCAAC‐3′; β‐actin, forward 5′‐CCCGAGCCGTGTTTCCT‐3′ and reverse 5′‐GTCCCAGTTGGTGACGATGC‐3′.

### 2.3 Cell proliferation assay

Cell proliferation was quantified using cell counting kit‐8 (CCK‐8) assay (Solarbio Science & Technology) as directed by the manufacturer's protocol. After transfection, cells were seeded into a 96‐well plate at a concentration of 500 cells per well. Cells were incubated with 10 μL of CCK‐8 solutions for 1.5 hours, and the absorbance was measured at 450 nm on a microplate reader. The OD value was checked every 24 hours.

### 2.4 Wound healing assays

The transfected cells were transferred into a 6‐well plate. Monolayer cells were scratched with a pipette tip and the wound closure was measured after incubation for 24 hours. The images were captured under a microscope. The relative migrated surface was analyzed using ImageJ software.

### 2.5 Transwell assay

Hundred‐microliter matrigel (BD) was added into the upper well of transwell chamber (Millipore) as directed by the manufacturer's protocol. Transfected cells were transferred into the upper well while 500 μL serum‐free medium was added into bottom well of transwell chamber. After incubated for 48 hours, cells were migrated to the lower chamber and stained with crystal violet for 10 minutes. Images were acquired and the number of cells was counted under a microscope. Each experiment was performed in triplicate.

### 2.6 Western blotting

The total protein was extracted from A549 cells using RIPA buffer containing protease inhibitors and separated through Sodium dodecyl sulfate‐polyacrylamide gel electrophoresis. Then, proteins were transferred to a PVDF membrane. After blocked with 5% fat‐free milk for 1 hour, the sample was incubated with primary antibodies for 1 hour and then with secondary antibodies for 1 hour. The following primary antibodies, namely anti‐Syncytin 1 (1:500), anti‐SP1 (1:1000) anti‐Active‐Caspase3 (1:1000), and anti‐Active‐Caspase9 (1:1000) were purchased from Abcam; anti‐Cyclin D1 (1:1000), anti‐Nusap1 (1:1000), anti‐CDK6 (1:1000), anti‐CDK4 (1:1000), anti‐Bcl2 (1:1000), anti‐Bax (1:1000), anti‐β‐catenin (1:1000), anti‐Vimentin (1:1000), anti‐E‐cadherin (1:1000), anti‐N‐cadherin (1:1000), anti‐P70 (1:1000), and anti‐GAPDH (1:5000) were purchased from Proteintech Group; anti‐p‐AKT (1:1000), anti‐p‐mTOR (1:1000), and anti‐p‐Erk1/2 (1:1000) were purchased from Cell Signaling Technology.

### 2.7 Flow cytometry analysis for cell cycle

A549 cells were seeded into the 6‐well plate and transfected with SP1 knockdown, Syncytin 1 knockdown, or Syncytin 1 overexpression vectors. After incubation for 24 hours, cells (1‐5 × 10^6^/mL) were fixed overnight with 70% ethanol, measured by 100 μL RNase at 37°C for 30 minutes, and stained with 400 μL PI (Roche) in dark environment. Red fluorescence was detected at 488 nm.

### 2.8 Flow cytometry analysis for the apoptosis

Annexin Ⅴ‐FITC/PI Kit (4Abio) was used for the analysis of cell apoptosis. After transfection for 48 hours, A549 cells were resuspended with Annexin V binding buffer to 1‐5 × 10^6^/mL. About 100 μL cell suspension was incubated with 5 μL Annexin V/FITC mix for 5 minutes. Flowjo software was used for the analysis of apoptosis cell numbers.

### 2.9 Statistical analyses

In this research, GrahpPad Prism 7.0 software was used for statistical analysis of results. The data were expressed as means ± SD. One‐way ANOVA analysis was used to assess differences between groups. Differences were considered statistically significant for values of *P* < .05.

## 3 RESULTS

### 3.1 Syncytin 1 knockdown inhibited the proliferation of NSCLC cells

**Figure 1:**
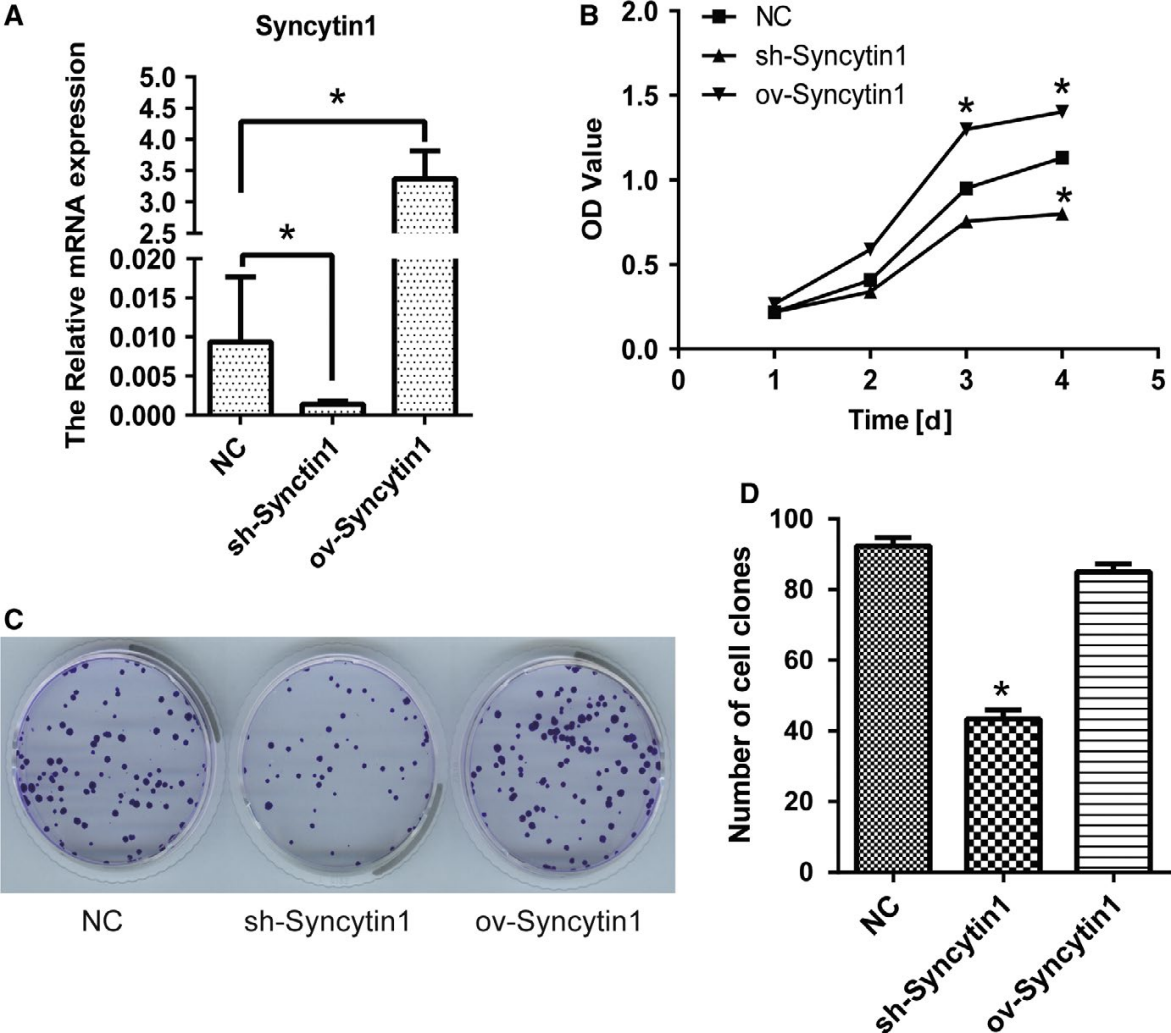
Syncytin 1 knockdown markedly inhibited the proliferation of NSCLC cells. A, qPCR was used for the detection of mRNA level of Syncytin 1 in A549 cells transfected with Syncytin 1 knockdown (sh‐Syncytin 1) or overexpression (ov‐Syncytin 1) plasmid, with the Lipofectamin™ 2000 as a negative control (NC). B, CCK‐8 assay was performed to determine the proliferation of A549 cells. The OD value was detected every 24 h after transfection. C, The proliferation of A549 cells was further detected by colony formation assay. Representative images were taken on 2 wk after transfection. D, The relative clone number of A549 cells was analyzed under the microscope. All excrements in this figure were performed in triplicate. *P* values were calculated using ANOVA. *P < .05 vs NC

We transfected Syncytin 1 knockdown and overexpression plasmids into NSCLC cell line A549 to generate the sh‐Syncytin 1 and ov‐Syncytin 1 cells, respectively. Results of quantitative real‐time PCR (qPCR) showed that the expression of Syncytin 1 was downregulated in sh‐Syncytin 1 cells, and markedly upregulated in ov‐Syncytin 1 cells (*P* < .05, Figure 1A) compared with the negative control (NC) cells.

We first investigated the effect of Syncytin 1 on cell proliferation using CCK‐8 assay. As shown in Figure 1B, the OD value declined significantly after the transfection with Syncytin 1 knockdown plasmid for 4 days compared with the NC cells (*P* < .05). After the transfection with Syncytin 1 overexpression plasmid for 3 days, the OD value increased markedly compared with the NC cells (*P* < .05). We further detected the proliferation of A549 cells by colony formation assay.

Two weeks after transfection, the relative clone number of A549 cells was analyzed under the microscope. From Figure 1C,D, Syncytin 1 underexpression inhibited the colony formation activity of A549 cells, but Syncytin 1 overexpression did not significantly alter the ability of colony formation of A549 cells. Therefore, we hypothesized that Syncytin 1 knockdown inhibited the proliferation of NSCLC cells.

### 3.2 Syncytin 1 knockdown blocked the cell cycle on G1 phase in NSCLC

**Figure 2:**
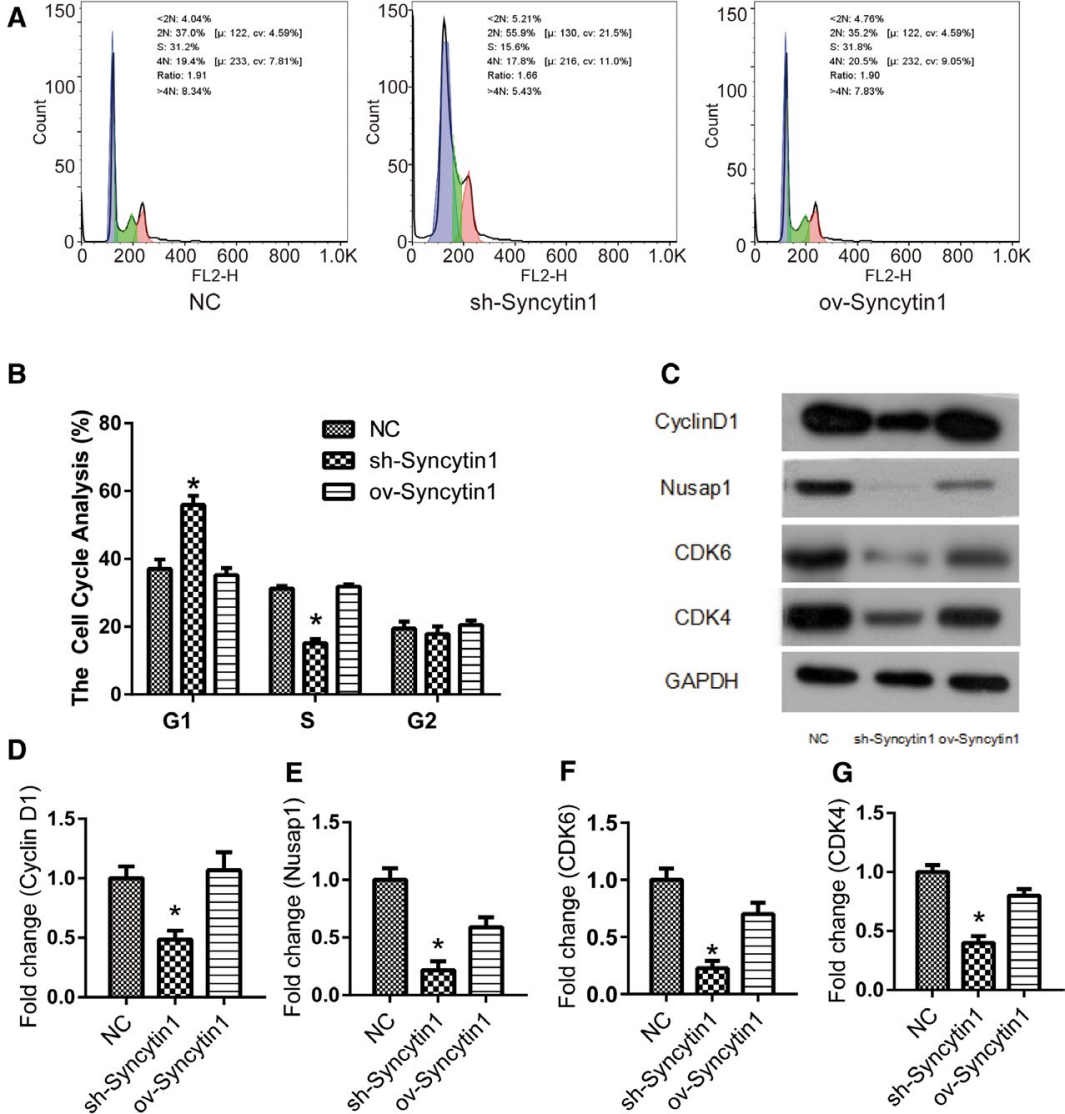
Syncytin 1 knockdown blocked the cell cycle on G1 phase. A and B, Flow cytometry analysis was performed to measure the cell cycle distribution of A549 cells and the results were analyzed by flowjo software. The percentage of cells on G1 phase markedly increased after the transfection of Syncytin 1 knockdown (sh‐Syncytin 1) plasmid. C, The protein levels of cell cycle relative genes, Nusap1 (D), Cyclin D1 (E), CDK6 (F), and CDK4 (G) were detected using western blotting and the results were measured by ImageJ software. All excrements in this figure were performed in triplicate. *P* values were calculated using ANOVA. *P < .05 vs NC

Flow cytometry analysis was performed to measure the cell cycle distribution of A549 cells. Compared with control cells, the percentage of cells on G1 phase markedly increased after the transfection of Syncytin 1 knockdown (sh‐Syncytin 1) plasmid (*P* < .05, Figure 2A,B). Furthermore, the percentage of cells on S phase both decreased after Syncytin 1 knockdown but without statistical differences. These data indicated that cells were blocked in G1 phase, resulting in a relative decrease in cell DNA synthesis and replication.

To further investigate the molecular mechanism of Syncytin 1 effect on cell cycle, the expression levels of related genes were detected by western blotting, including Nusap1, Cyclin D1, CDK6, and CDK4. As shown in Figure 2C‐G, the expression levels of Nusap1, Cyclin D1, CDK6, and CDK4 were all downregulated by Syncytin 1 knockdown (*P* < .05). These proteins were important factors in cell cycle regulation, promoting and coordinating cell cycle progression. Therefore, we speculated that Syncytin 1 prevented cells from entering the cell cycle by inhibiting the expression of these proteins.

### 3.3 Syncytin 1 knockdown promoted the apoptosis of NSCLC cells

**Figure 3:**
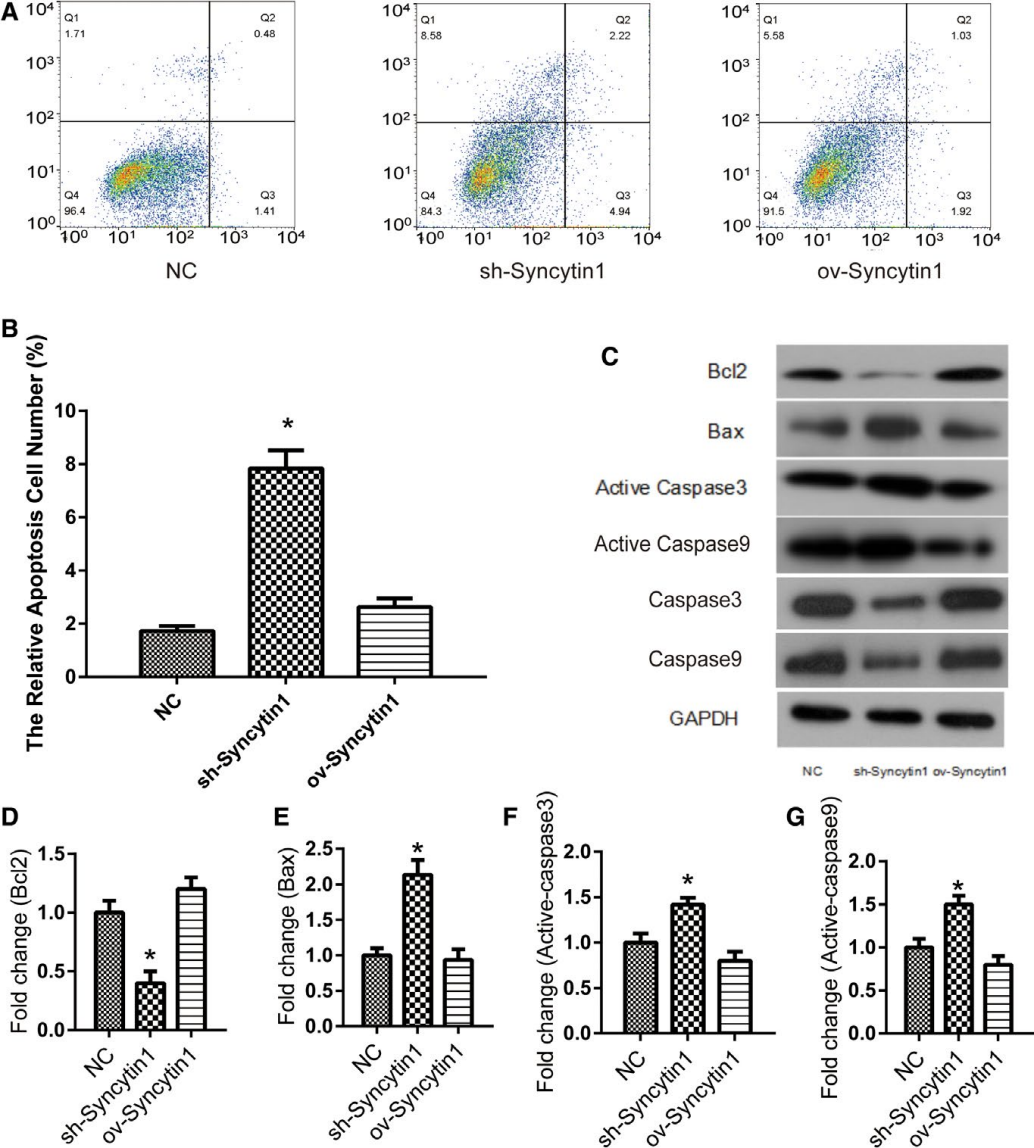
Syncytin 1 underexpression promoted the apoptosis of NSCLC cells. A and B, Flow cytometry analysis was performed to measure the apoptosis of A549 cells. The relative apoptosis cell number was analyzed using flowjo software. C, The protein levels of apoptosis relative genes, Bcl2 (D), Bax (E), Active‐Caspase3 (F), and Active‐Caspase9 (G) were detected using western blotting and the results were measured by ImageJ software. All excrements in this figure were performed in triplicate. *P* values were calculated using ANOVA. *P < .05 vs NC

When cells are arrested in G1 phase, a series of repair reactions can be triggered, and if the repair fails, the mechanism of apoptosis is triggered. Therefore, we further investigated that whether Syncytin 1 knockdown could induce the apoptosis in NSCLC cells. As shown in Figure 3A,B, flow cytometry analysis was performed to measure the apoptosis of cells transfected with Syncytin 1 knockdown or overexpression plasmids (*P* < .05). The relative apoptosis cell number increased markedly after the transfection of Syncytin 1 knockdown plasmid.

To investigate the mechanism of Syncytin 1 effect on cell apoptosis, the protein levels of apoptosis relative genes, Bcl2, Bax, Active‐Caspase3, and Active‐Caspase9 were detected using western blotting. As shown in Figure 3C‐G, the levels of apoptosis factor, Active‐Caspase9, Active‐Caspase3, and Bax, were markedly upregulated after the suppression of Syncytin 1. In contrary, the expression of apoptosis inhibitory factor, Bcl‐2, was downregulated in Syncytin 1 knockdown cells. These data proved that Syncytin 1 underexpression promoted the apoptosis of NSCLC cells.

### 3.4 Syncytin 1 knockdown declined the migration and invasion of NSCLC cells

**Figure 4:**
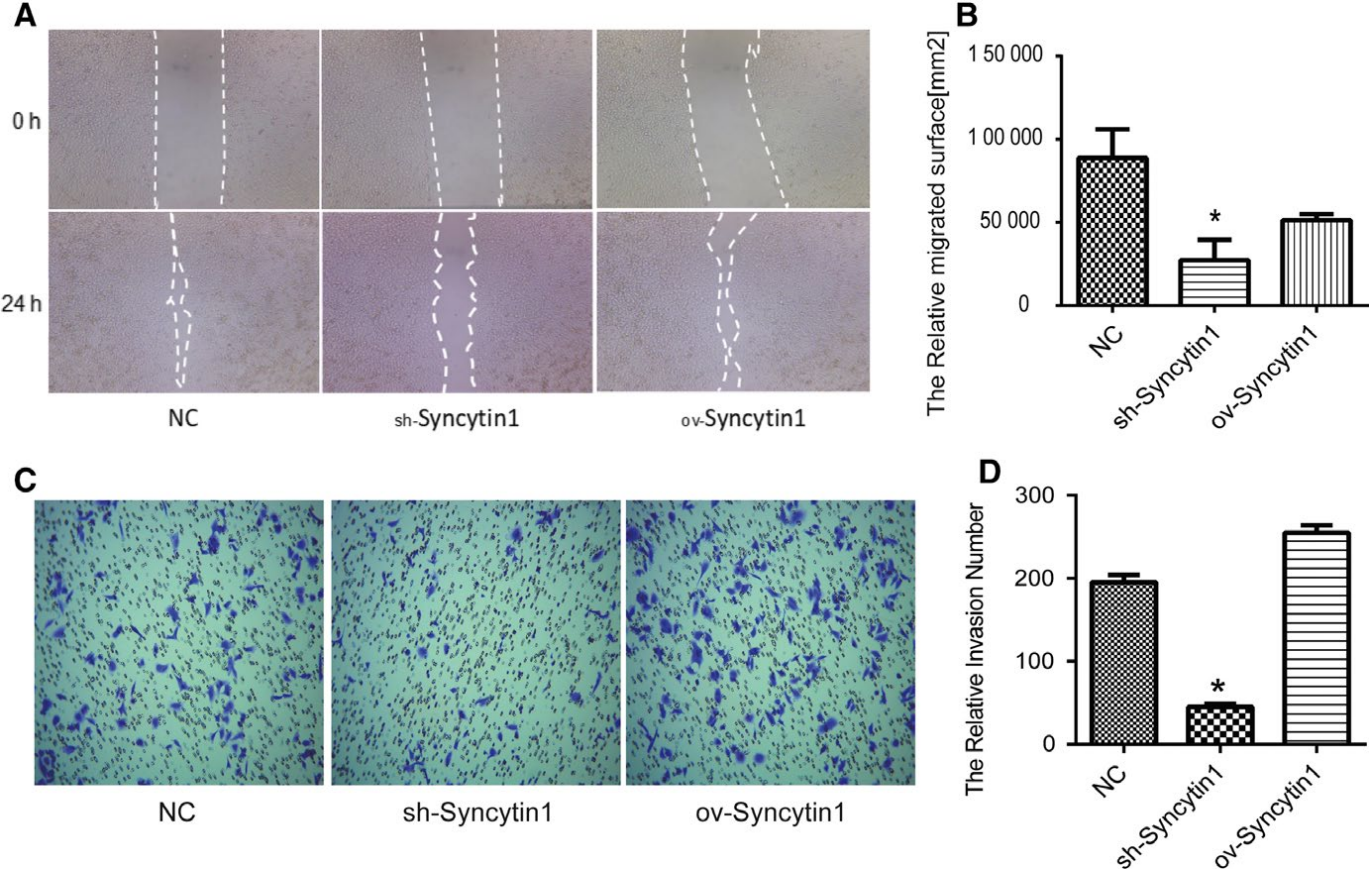
Syncytin 1 underexpression declined the migration and invasion of NSCLC cells. A and B, After transfection with Syncytin 1 knockdown (sh‐Syncytin 1) or overexpression (ov‐Syncytin 1) plasmid, cell migration was detected using wound healing assay. The relative migrated surface was analyzed using ImageJ software after the transfection for 24 h. C and D, The invasion of A549 cells was determined using matrigel invasion chambers after transfection for 48 h. The relative invasion number was counted in five random fields. All excrements in this figure were performed in triplicate. *P* values were calculated using ANOVA. *P < .05 vs NC

The role of Syncytin 1 on cell migration and invasion was investigated by wound healing and matrigel transwell respectively. From the results of wound healing assay, the relative migrated surface reduced significantly after transfection with Syncytin 1 knockdown plasmids (*P* < .05, Figure 4A,B). Moreover, Syncytin 1 knockdown also inhibited the relative invasion cell number in A549 cells according to results of matrigel transwell (*P* < .05, Figure 4C,D). However, Syncytin 1 overexpression did not alter the ability of the migration and invasion of A549 cells. We predict that the effect of Syncytin 1 on cell function may be limited by a downstream target or signaling pathway. Taken together, Syncytin 1 knockdown inhibited the migration and invasion of human NSCLC cells.

### 3.5 Syncytin 1 knockdown reversed epithelial‐mesenchymal transition progress via inhibiting the Akt and Erk1/2 signaling pathway

**Figure 5:**
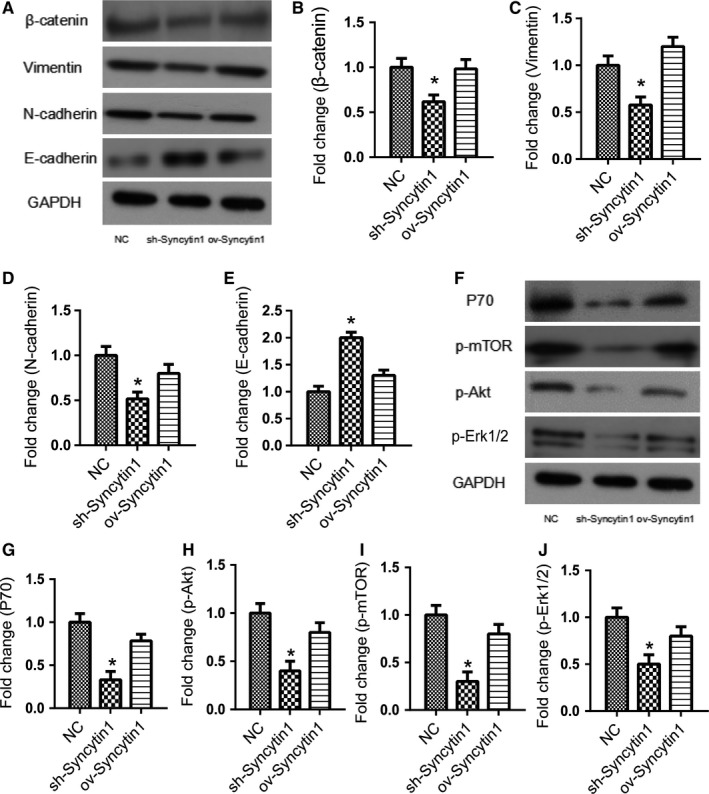
Syncytin 1 underexpression reversed EMT progress and inhibited the Akt signaling pathway. A, The protein levels of EMT relative genes, β‐catenin (B), Vimentin (C), N‐cadherin (D), and E‐cadherin (E) were detected using western blotting. F, The protein levels of the Akt and Erk signaling pathways relative genes, P70 (G), p‐Akt (H), p‐mTOR (I), and p‐Erk (J) were detected using western blotting. The results were measured by ImageJ software. All excrements in this figure were performed in triplicate. *P* values were calculated using ANOVA. *P < .05 vs NC

To elucidate the molecular mechanism of the effect of Syncytin 1 on migration and invasion of NSCLC cells, we detected the expression levels of epithelial‐mesenchymal transition (EMT)‐related genes by western blot. EMT plays an important role in the motility of tumor cells and is a vital process in the origin and metastasis of solid tumors.^15^ The protein levels of EMT relative genes, β‐catenin, Vimentin, N‐cadherin, and E‐cadherin were detected using western blotting. As shown in Figure 5A‐E, Syncytin 1 underexpression suppressed the expressions of N‐cadherin, β‐catenin, and Vimentin, and promoted the expression of E‐cadherin (*P* < .05).

Studies have shown that Akt and Erk pathways, as a key signaling pathway regulating cell proliferation, migration, and EMT progress, can be significantly activated in NSCLC.^16^, ^17^ Thus, we performed the western blotting to detect the protein levels of the Akt and Erk signaling pathways relative genes, P70, p‐Akt, p‐mTOR, and p‐Erk. As shown in Figure 5F‐J, the phosphorylation levels of Akt, mTOR, and Erk were all decreased in Syncytin 1 knockdown cells in comparison to the NC cells (*P* < .05), suggesting the inhibition of Akt and Erk signaling pathways. Taken together, our results proved that Syncytin 1 knockdown reversed EMT progress via inhibiting the Akt and Erk1/2 signaling pathway.

### 3.6 SP1 inhibited the expression of Syncytin 1 in NSCLC cells

**Figure 6:**
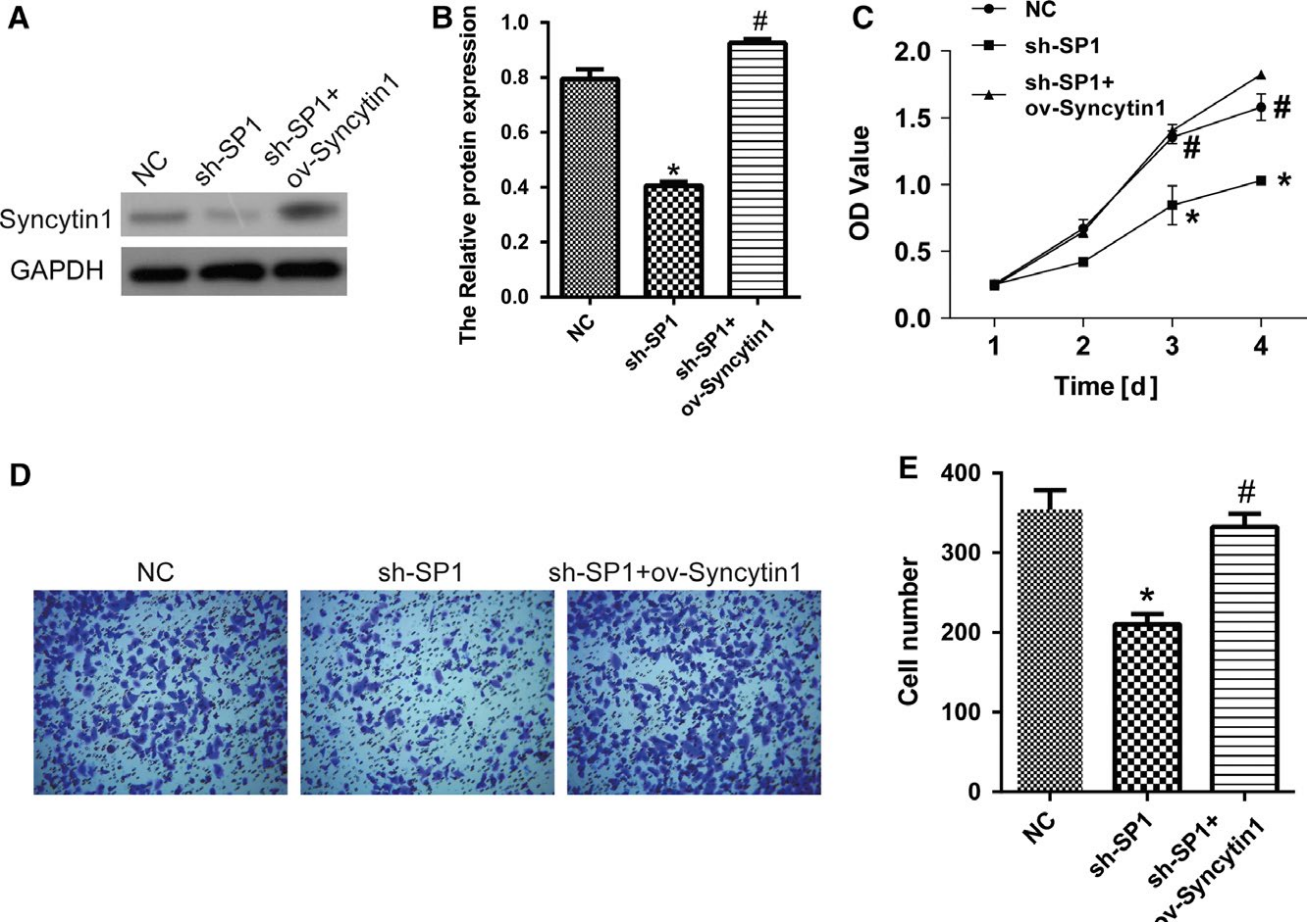
SP1 inhibited the expression of Syncytin 1 and Syncytin 1 blocked the decline of proliferation and metastasis induced by SP1 knockdown. A and B, Syncytin 1 expression was detected by western blotting in cells transfected with SP1 knockdown plasmid (sh‐SP1) or with SP1 knockdown and Syncytin 1 overexpression plasmids (sh‐SP1+ov‐Syncytin 1). C, The proliferation of sh‐SP1 and sh‐SP1+ov‐Syncytin 1 cells was determined by CCK‐8 assay. The OD value was detected every 24 h after transfection. D and E, The invasion of A549 cells was determined using matrigel invasion chambers after transfection for 48 h. The relative invasion number was counted in five random fields. All excrements in this figure were performed in triplicate. *P* values were calculated using ANOVA. *P < .05 vs NC; ^#^ *P* < .05 vs sh‐SP1

It has been reported that transcription factor SP1 induces human Syncytin promoter activity and is critical to its cell‐specific expression.^18^ Therefore, in this study we attempt to evaluate whether SP1 can regulate the expression of Syncytin1 in lung cancer cells. SP1 knockdown plasmid was transfected into A549 cells to generate sh‐SP1 cells. The result of western blotting proved that Syncytin 1 expression was inhibited by SP1 knockdown (sh‐SP1), which was reversed by Syncytin 1 overexpression (sh‐SP1+ov‐Syncytin 1), indicating that SP1 inhibited Syncytin 1 expression in NSCLC cells (*P* < .05, Figure 6A,B).

### 3.7 Syncytin 1 blocked the inhibition of cell growth induced by SP1 knockdown in NSCLC cells

**Figure 7:**
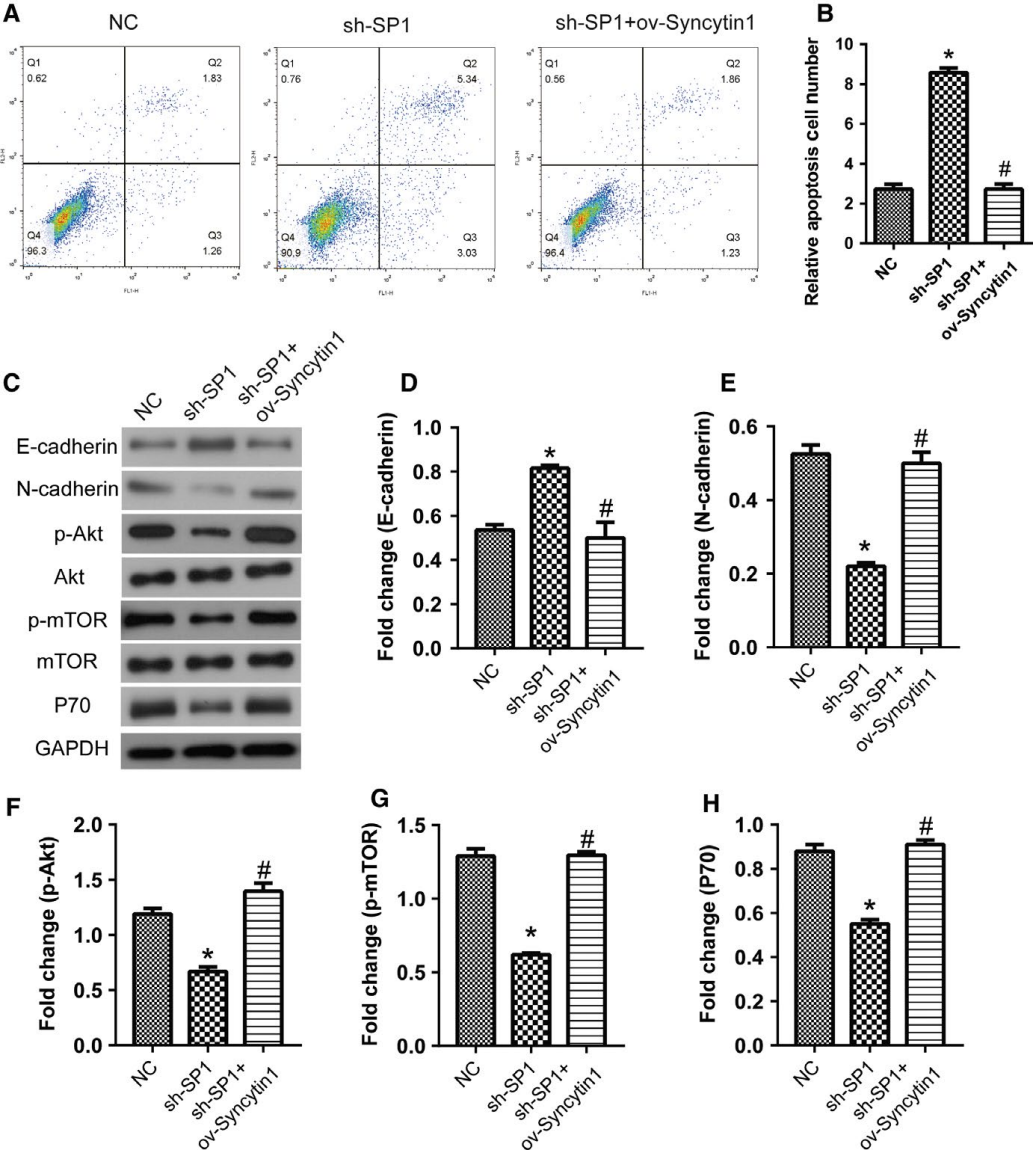
The overexpression of Syncytin 1 inhibited the increase in the apoptosis induced by SP1 knockdown in NSCLC cells. A and B, Flow cytometry analysis was performed to measure the apoptosis of A549 cells. The relative apoptosis cell number was analyzed using flowjo software. C, The protein levels of E‐cadherin (D), N‐cadherin (E), p‐Akt (F), p‐mTOR (G), and P70 (H) were detected using western blotting. The results were measured by ImageJ software. All excrements in this figure were performed in triplicate. *P* values were calculated using ANOVA. *P < .05 vs NC; ^#^ *P* < .05 vs sh‐SP1

We further investigated the proliferation of sh‐SP1 and sh‐SP1+ov‐Syncytin 1 cells by CCK‐8 assay. After transfection for 3 days, the OD value declined markedly in sh‐SP1 cells compared with the control cells, and increased significantly in sh‐SP1+ov‐Syncytin 1 cells compared with sh‐SP1 cells (*P* < .05, Figure 6C). In the matrigel transwell assay, consistent results were also observed. The relative invasion number decreased in sh‐SP1 cells compared with the control cells, and increased significantly in sh‐SP1+ov‐Syncytin 1 cells compared with sh‐SP1 cells (*P* < .05, Figure 6D,E). Furthermore, the relative apoptosis cell number detected by flow cytometry analysis was increased in sh‐SP1 cells compared with the control cells, and decreased markedly in sh‐SP1+ov‐Syncytin 1 cells compared with sh‐SP1 cells (*P* < .05, Figure 7A,B). These results proved that Syncytin 1 blocked the inhibition of cell growth induced by SP1 knockdown, suggesting that Syncytin 1 activity was promoted by transcription factor SP1 in NSCLC.

The results of western blotting showed that SP1 inhibited the expressions of N‐cadherin, p‐Akt, p‐mTOR, and P70, but promoted the E‐cadherin expression, indicating that Syncytin 1 could block the revers of EMT and inhibition of the Akt signaling pathway induced by SP1 knockdown (*P* < .05, Figure 7C,H). Taken together, we hypothesized that SP1 promoted Syncytin1 expression and knockdown of SP1/Syncytin1 axis inhibited the proliferation and metastasis through the AKT signaling pathway in human NSCLC cells.

## 4 DISCUSSION

In the present study, we confirmed that interfering of Syncytin 1 inhibited the proliferation, metastasis, and promoted apoptosis of lung cancer cells by reversing EMT pathway through a series of in vitro experiments, which was regulated by transcription factor SP1.

First, our results proved that Syncytin 1 knockdown inhibited the proliferation, blocked the cell cycle on G1 phase, and downregulated the expressions of Nusap1, Cyclin D1, CDK6, and CDK4 in A549 cells. There are key regulatory checkpoints in different phases of cell cycle. Different cyclin and cyclin‐dependent kinase (CDK) complexes are crucial to these checkpoints.^19^, ^20^ The phosphorylation of the different substrates caused by CDK‐cyclin complexes results in a series of cascades that ultimately regulate the expression of proliferation‐related genes, and the concentration and activity of different CDK‐cyclin complexes in cells are regulated by other components.^19^ The most important checkpoint is between G1 and S phase, which is called restriction point in mammalian cells.^20^ At this point, cells integrate and transmit complex intracellular and extracellular signals, including various growth factors, mitogens in the serum, and DNA damage, to determine whether cells enter the division, programmed death or static G0 phase.^21^ The uncontrolled proliferation caused by abnormal checkpoint between G1 and S is crucial for the progress of tumors.^22^, ^23^ Furthermore, we found that Syncytin 1 knockdown induces cell apoptosis by promoting the expressions of Bax, Active‐Caspase3, and Active‐Caspase9 in A549 cells. Therefore, we hypothesized that Syncytin 1 knockdown blocked cells in G1 phase and induced apoptosis by inhibiting cyclin D1‐CDK4/6 pathway, thus inhibiting the proliferation of cancer cells.

Next, we found that Syncytin 1 knockdown inhibited the migration and invasion of A549 cells according to the results of wound healing and matrigel transwell assays. The detachment and migration of tumor cells from the primary location is an important part of lung cancer metastasis, which is closely related to the decrease in adhesion ability between tumor cells, suggesting that knockdown of Syncytin 1 can inhibit the metastasis of NSCLC.^24^ In addition, Syncytin 1 underexpression also suppressed the expressions of N‐cadherin, β‐catenin, and Vimentin, and promoted the expression of E‐cadherin. These data indicated that Syncytin 1 knockdown inhibits the metastasis of NSCLC through reversing the EMT process.

Then, the results of western blotting showed that Syncytin 1 knockdown could inhibit the activity of Akt and Erk1/2 signaling pathways. Studies have shown that about 50% to 70% of NSCLC patients have abnormal phosphorylation of Akt and mTOR, which plays an important role in cell proliferation, survival, chemotherapy, and radiotherapy resistance.^25^ The downstream genes of Akt signaling pathway, Cyclin D1 and P70, were also inhibited by Syncytin 1 knockdown, which further confirmed the signaling pathway by which Syncytin 1 knockdown inhibited the tumor progression.

Although knockdown of Syncytin 1 inhibited the proliferation and metastasis of NSCLC cells, our results showed that overexpression of Syncytin 1 did not promote the growth of cancer cells. We speculate that the effect of Syncytin 1 reaches a threshold, which may be limited by its downstream targets or signaling pathways. However, its specific mechanism still needs further study.

Moreover, our data proved that knockdown of transcription factor SP1 inhibited the expression of Syncytin 1 in A549 cells. SP1 is a nuclear transcription factor that plays an important regulatory role in mammalian gene expression. SP1 affects the growth and metastasis of tumors by regulating the expressions of tumor‐related genes.^26^, ^27^, ^28^ It can bind to specific sites on the promoter of vascular endothelial growth factor (VEGF) or VEGF receptor, which further promotes the progress of tumors.^29^ SP1‐regulated tumor‐related genes are involved in a variety of cell functions, including cell cycle (Cyclin D1, TGF‐α, E2F1),^30^, ^31^ apoptosis (Bax, Bcl2),^32^, ^33^ tumor metastasis (TGF‐β, MMP2)^34^ and angiogenesis (VEGF).^35^ According to our results, Syncytin 1 may be one of the targets of SP1 in regulating the function of cancer cells. In addition, SP1 is one of the substrates of Erk1/2 signaling pathway, and activated by phosphorylation of Erk1/2.^36^ We hypothesize that Erk1/2 phosphorylation upregulates the expression of Syncytin‐1 by activating SP1, thereby promoting tumor growth. Excessive expression of Syncytin 1 may form a feedback inhibition, thereby limiting its growth‐promoting effect on tumor cells. This will also be the focus of our future research.

In conclusion, Syncytin 1 was upregulated by transcription factor SP1, and its knockdown blocks cell proliferation, migration and invasion, and induced cell cycle arrest and apoptosis by inhibiting the activity of Akt/mTOR and Erk1/2 signaling pathways in NSCLC. Our results contribute to expanding the knowledge of Syncytin 1 functions in promoting tumor progression, and provide a potential target for the clinical treatment of human NSCLC.

## CONFLICT OF INTEREST

The authors declare no conflict of interest.
